# In severe ADNC, hippocampi with comorbid LATE-NC and hippocampal sclerosis have substantially more astrocytosis than those with LATE-NC or hippocampal sclerosis alone

**DOI:** 10.1093/jnen/nlad085

**Published:** 2023-11-03

**Authors:** Dana M Niedowicz, Yuriko Katsumata, Peter T Nelson

**Affiliations:** University of Kentucky, Lexington, Kentucky, USA; University of Kentucky, Lexington, Kentucky, USA; University of Kentucky, Lexington, Kentucky, USA

**Keywords:** ADRD, Astrocytes, Digital pathology, Gliosis, Hippocampal sclerosis, Inflammation, ScanScope

## Abstract

Limbic-predominant age-related TDP-43 encephalopathy neuropathologic change (LATE-NC) and hippocampal sclerosis of aging (HS-A) pathologies are found together at autopsy in ∼20% of elderly demented persons. Although astrocytosis is known to occur in neurodegenerative diseases, it is currently unknown how the severity of astrocytosis is correlated with the common combinations of pathologies in aging brains. To address this knowledge gap, we analyzed a convenience sample of autopsied subjects from the University of Kentucky Alzheimer’s Disease Research Center community-based autopsy cohort. The subjects were stratified into 5 groups (n = 51 total): pure ADNC, ADNC + LATE-NC, ADNC + HS-A, ADNC + LATE-NC + HS-A, and low-pathology controls. Following GFAP immunostaining and digital slide scanning with a ScanScope, we measured GFAP-immunoreactive astrocytosis. The severities of GFAP-immunoreactive astrocytosis in hippocampal subfield CA1 and subiculum were compared between groups. The group with ADNC + LATE-NC + HS-A had the most astrocytosis as operationalized by either any GFAP+ or strong GFAP+ immunoreactivity in both CA1 and subiculum. In comparison to that pathologic combination, ADNC + HS or ADNC + LATE-NC alone showed lower astrocytosis. Pure ADNC had only marginally increased astrocytosis in CA1 and subiculum, in comparison to low-pathology controls. We conclude that there appeared to be pathogenetic synergy such that ADNC + LATE-NC + HS-A cases had relatively high levels of astrocytosis in the hippocampal formation.

## INTRODUCTION

Limbic-predominant age-related TDP-43 encephalopathy neuropathologic change (LATE-NC) and hippocampal sclerosis of aging (HS-A) pathologies are found together at autopsy in up to one-fourth of elderly demented persons (1–20). The nomenclature is potentially confusing because the term hippocampal sclerosis is used to refer to other conditions (e.g. epilepsy and transient anoxia) that are completely different in pathogenesis and clinical implications (21). There also is an imperfect overlap between the terminology used in the neuropathological and clinical radiology fields.

From a neuropathological perspective, the cardinal feature of LATE-NC is temporal lobe-predominant TDP-43 proteinopathy (21), whereas HS-A is defined as excess neuronal loss and astrocytosis in the hippocampal formation (22). The association between LATE-NC and HS-A was first reported by Amador-Ortiz et al (11). LATE-NC + HS-A pathology has a strong negative association with cognitive impairment, even after factoring in other pathologies (1, 23, 24). Despite progress in the field, there remain many unanswered questions about pathological combinations that include ADNC and LATE-NC + HS-A. Not all LATE-NC cases have HS-A (or vice versa) (4, 9), and the genetic contributors to LATE-NC and HS-A may be separate (25). Even among the cases with LATE-NC, the presence of HS-A is independently associated with added cognitive impairment (23, 26). Hence, it may be instructive to study the cases where LATE-NC and HS-A are disassociated.

HS-A itself—usually a triad of neuronal cell loss, TDP-43 pathology, and astrocytosis—has been studied in recent years (19, 20, 26–29). The presence and severity of neuronal loss and TDP-43 pathology in HS-A have been analyzed previously (16, 28, 30, 31). By contrast, the relevance of astrocytosis to the pathogenesis is currently unknown.

Astrocytes and their pathologies are foci of interest in the dementia research world (32–35). The presence of astrocytes in and around pathologic lesions may correspond to a reactive change or the cells (via inflammatory or other mechanisms) may themselves participate in the pathogenesis; these possibilities are not mutually exclusive. Autopsy-based studies provide a perspective for understanding some aspects of the disease-associated phenomenology. Whereas neuropathologic evaluations cannot necessarily indicate the exact mechanisms involved, they can help address basic questions about the astrocytosis detected in different disease states.

Here, we studied the severity of GFAP-immunoreactive astrocytosis in ADNC, with or without LATE-NC, and/or comorbid HS-A pathology. Research participants were recruited into the University of Kentucky Alzheimer’s Disease Research Center (UK-ADRC) community-based cohort, which has been described in detail (36).

## MATERIALS AND METHODS

Adult volunteers recruited into the UK-ADRC community-based cohort (36, 37) (most age 70 years and above) agreed to be followed annually for cognitive, physical, and neurological examination and to donate their brain after death. Protocols and informed consent procedures were approved by the University of Kentucky Institutional Review Board. Certain exclusion criteria were applied at recruitment (e.g. substance use disorder history and severe neuropsychiatric disorders) (36, 37). For the present study, we excluded individuals with rare conditions recruited from a memory disorders clinic; frontotemporal lobar degeneration, amyotrophic lateral sclerosis, multiple system atrophy, chronic traumatic encephalopathy, and triplet repeat disorders were thus not included.

Samples of hippocampus were fixed in 10% phosphate-buffered formalin, embedded in paraffin, and sectioned to 8-µm thickness using a microtome. Slides were deparaffinized in xylene, followed by decreasing concentrations of ethanol. Neurofibrillary pathology/phospho-Tau (pTau) pathology was operationalized using the PHF-1 antibody (gift of Dr. Peter Davies) as described previously (38). Severe ADNC was as defined by Montine et al (22); practically, this indicates Braak NFT stages V or VI. Phospho-TDP-43 pathology was assessed by immunostaining using the 1D3 antibody (39), using methods described previously (40). All LATE-NC cases corresponded to LATE-NC Stage 2 (41). As to the operationalization of HS-A, we followed the criteria described in (22).

For digital quantification of ADNC in the hippocampal formation, immunostained slides were loaded into an Aperio (now Leica) ScanScope slide scanner. Slides were scanned at 40× magnification, the data were stored on a dedicated server, and analyses were performed as previously described in detail (42, 43). For pTau digital quantification analyses, measurements of lesion burden were generated representing densities of tau-immunoreactive NFTs that were detected. Batch normalization was not performed. The methodologies, algorithm inputs, and thresholding parameters were applied as previously reported in detail (42, 43).

For digital quantification of GFAP-immunoreactive astrocytosis in the hippocampal formation, hippocampal sections were cut onto slides, immunohistochemically stained for GFAP, loaded into a ScanScope slide scanner and amounts of GFAP immunoreactivity were quantified in CA1 and subiculum. Prior to GFAP immunohistochemistry, antigen retrieval was performed by boiling the slides in citrate buffer (pH 6) for 6 minutes, followed by formic acid (3 minutes, room temperature). Endogenous peroxidases were neutralized with H_2_O_2_ (3% in methanol, 30 minutes, room temperature). Slides were then blocked in normal horse serum (15%, 1 hour, room temperature) and incubated with the anti-GFAP primary antibody (Santa Cruz #sc-33673, 1:200 in 15% horse serum) overnight at 4°C. After washing, slides were incubated with biotinylated horse anti-mouse secondary antibody (Vector Laboratories; 1 hour, room temperature), followed by ABC reagent (Vector Laboratories, 1 hour, room temperature). Slides were developed with Vector Nova Red HRP substrate, counterstained with hematoxylin, dehydrated, and cover-slipped with a toluene-based mounting media (Fisher Scientific). Analyses were performed as previously described (42, 43). Four 1-mm^2^ regions of interest (ROIs) were drawn in the CA1 and subiculum regions of each slide. Counts were generated by a researcher that was blind to the neuropathology other than what could easily be observed on a GFAP-immunostained slide. The Positive Pixel Count algorithm was used to analyze each of these ROIs, setting the intensity thresholds to identify only positively stained cells. These thresholds were kept constant for all slides analyzed. Data were expressed as GFAP positivity (% of total negative + positive staining) and the numbers were averaged between the 4 ROIs for each of the brain regions analyzed.

## RESULTS

**Figure 1.**
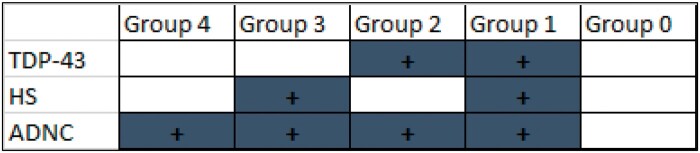
Schematic representation of the 5 groups of cases as defined by neuropathologic observations.

**Figure 2.**
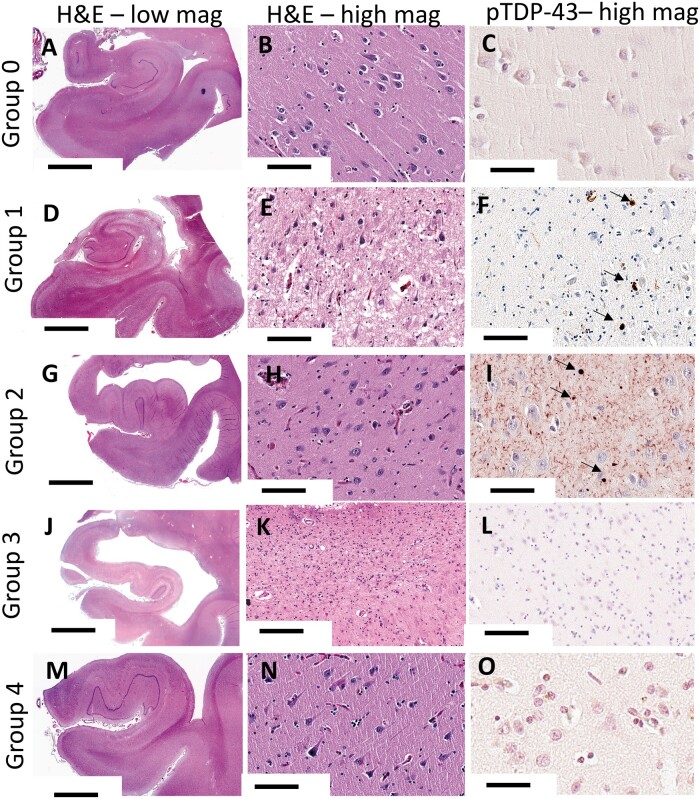
Photomicrographs depicting histopathology and phospho-TDP-43 (pTDP-43) immunohistochemistry in representative portions of the hippocampi from each of 5 groups. **(A**, **D**, **G**, **J**, and **M)** Low-magnification photomicrographs of anterior hippocampi are shown. **(B**, **E**, **H**, **K**, and **N)** Higher-magnification photomicrographs enable visualization of cellular constituents and neuropil rarefaction. Overall, there was slightly more neuropil rarefaction in Group 1 **(E)**, but this was not always the case **(C**, **F**, **I**, **L**, and **O)**. pTDP-43 immunohistochemical photomicrographs showing neuronal cytoplasmic pTDP-43 inclusions [arrows in **(F)** and **(I)**]. Scales bars: **A**, **G**, and **M** = 6 mm; **B**, **E**, **H**, **K**, and **N** = 200 mm; **C** and **O** = 60 μm; **F**, **L**, and **I** = 120 μm; and **D** and **J** = 5 mm.

**Figure 3.**
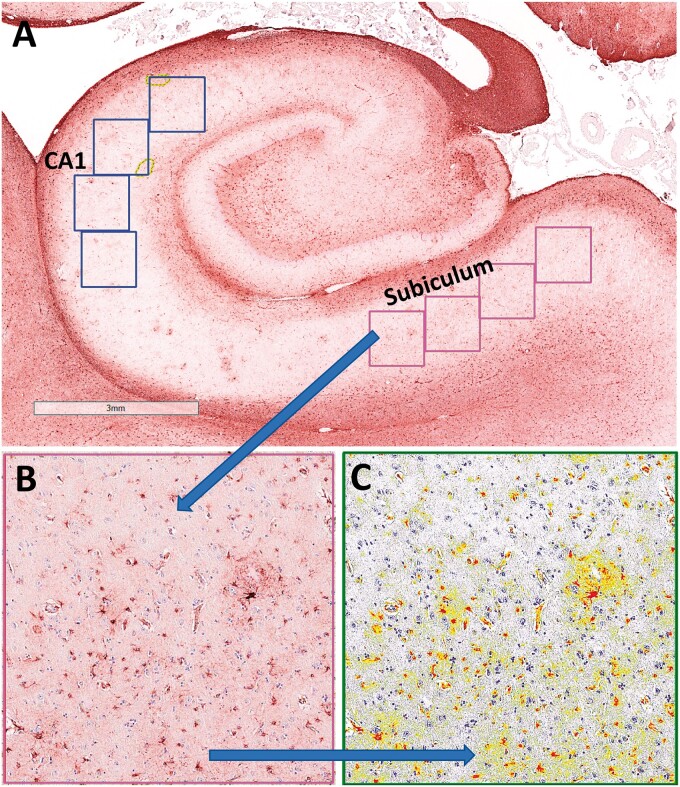
Digital photomicrograph of GFAP immunoreactivity in a control human hippocampus. **(A)** Blue boxes are CA1, and pink boxes are subiculum. **(B** and **C)** The leftmost subiculum box is shown at higher power. Panel **(B)** depicts the GFAP immunoreactivity and panel **(C)** is false-colored to indicate strong GFAP immunopositivity (red) and other GFAP immunopositivity (yellow) that were registered and quantified for each case.

For the purpose of the current study, the cases were stratified by the presence and combinations of detected neuropathologies. Information about the included subjects—a convenience sample of n = 51 autopsied subjects—is shown in the Table. One group of individuals lacked LATE-NC, severe ADNC, or HS-A (this was designated Group 0; n = 8). Otherwise, the cases included in the current study had severe ADNC: Group 1 (LATE-NC+HS-A; n = 10); Group 2 (LATE-NC no HS-A; n = 9); Group 3 (HS-A no LATE-NC; n = 11); and Group 4 with relatively pure ADNC (n = 13). Those included with LATE-NC were all LATE-NC Stage 2 (41). To be clear, the term “pure ADNC,” in this context, only means that the cases lack LATE-NC or HS-A; many of these would be expected to have other pathology, especially vascular pathologies that are very common in aging brains. These groups are depicted schematically in Figure 1. Photomicrographs of representative cases are shown in Figures 2 and 3.

**TABLE.** Case Categories and Characteristics of Included Subjects

	Group #				
	0	1	2	3	4
n	8	10	9	11	13
Age at death (years)	82.0	79.5	86.9	85.1	87.1
% male	75	80	44	64	46
Final MMSE score, avg	28.8	6.6	19.5	14.3	19.4
*APOE* ε4 (%)	28.6	50.0	37.5	70.0	46.2
NFT density,^a^ CA1	4.4	81.7	55.3	82.0	51.0
NFT density,^a^ subiculum	3.9	99.7	42.4	80.0	50.8
LATE-NC Stage 2	None	All	All	None	None
HS-Aging pathology	None	All	None	All	None
Severe ADNC (Braak Stages V/VI)	None	All	All	All	All
Clinical history of stroke, n (%)	1 (12.5%)	4 (40%)	0	1 (9.1%)	0
Clinical history of seizures, n (%)	0	0	1 (11.1%)	2 (9.1%)	3 (23.1%)

a Counted as described in Neltner et al (43) and described in Materials and Methods.

**Figure 4.**
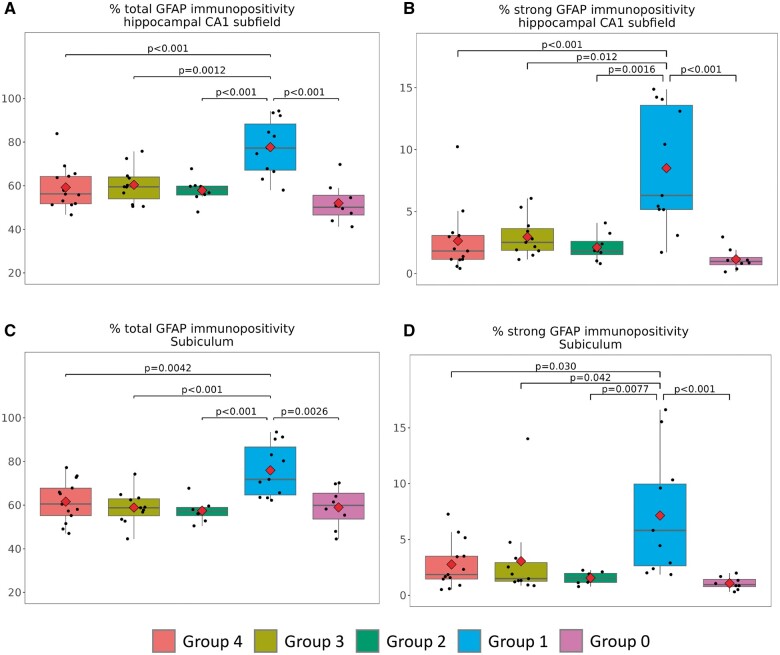
Digital neuropathologic assessment of GFAP immunoreactivity in 5 groups. See Figure 1 and the Table for description of group characteristics. GFAP immunoreactivity was quantified in CA1 of the hippocampus **(A** and **B)** and subiculum **(C** and **D)** and operationalized as total GFAP immunoreactivity **(A** and **C)** and strong GFAP immunoreactivity **(B** and **D)**. Note that Group 1 (ADNC + LATE-NC + HS) had consistently the most GFAP immunoreactivity. Statistical tests and p-values reflect findings using ANOVA.

After delineating the groups according to the neuropathological patterns, the study was oriented toward evaluating whether there were group-level differences in the detected severities of astrocytosis, (operationalized according to GFAP-immunoreactive signal), in 2 portions of the hippocampal formation: CA1 subfield and subiculum. The results for both regions were assessed based on the study of all GFAP immunoreactivity detected (Fig. 4A, C) and, separately, the most intense levels of GFAP immunoreactivity detected (Fig. 4B, D).

Among the 5 pathology-based groups, the group with ADNC + LATE-NC + HS-A had the most severe astrocytosis in both CA1 and subiculum. By contrast, “pure” ADNC, ADNC + LATE-NC, and ADNC + HS-A had comparable amounts of astrocytosis as operationalized by either any GFAP+ or strong GFAP+ immunoreactivity in CA1 and subiculum of the hippocampal formation. The controls (lacking ADNC, LATE-NC, or HS-A) had marginally lower astrocytosis. After adjusting for sex, age at death, *APOE* ε4 carrier or not (0/1), the trend for difference between Group 3 versus Group 1 in strong GFAP immunoreactive subiculum was no longer statistically significant (p = 0.069 for adjusted model). Other statistical significance remained even after adjusting for those covariates. In other words, following adjustment for those covariates, the differences in strong GFAP immunoreactivity (i.e. highest in ADNC + LATE-NC + HS-A) all were statistically significant for CA1 and for overall GFAP immunoreactivity in both subiculum and CA1.

## DISCUSSION

In the present study of autopsied individuals from a community-based cohort that was stratified into 5 case categories, those with the combination of severe ADNC, LATE-NC, and HS-A had the most astrocytosis in the hippocampal CA1 and subiculum. After adjusting for covariates such as *APOE* genotypes, the trend for association between strong GFAP immunoreactivity and pathology-based group results, comparing ADNC + LATE-HS + HS-A and ADNC + HS-A in subiculum, was no longer statistically significant (p = 0.069). However, differences in this measure were statistically significant for CA1 and were also statistically significant for overall GFAP immunoreactivity in both subiculum and CA1. Thus, overall, there was a robust trend for ADNC + LATE-NC + HS-A to have the most GFAP-immunoreactive astrocytosis. Hippocampi with relatively “pure” ADNC, or ADNC + LATE-NC, had lower levels of GFAP+ gliosis in CA1 and subiculum regions. These results shed light on the fact that astrocytosis may be an important component of the pathogenesis of ADNC + LATE-NC + HS-A, in comparison to cases with “pure” LATE-NC or HS-A without LATE-NC.

It has been shown previously that astrocytes may participate in both reactive and/or disease-driving mechanisms in brains with TDP-43 pathology (44–47). For example, Alexander disease, driven by mutations in *GFAP* gene with pathological astrogliosis and neurodegeneration, is commonly associated with TDP-43 proteinopathy (48). Nonetheless, both the mechanisms and the basic phenomenology (anatomical distribution and severity) of astrocytosis in LATE-NC pathology (with or without comorbid ADNC) and HS-A are incompletely understood.

Although there was no pathological-radiographical correlation in the present study, our findings have implications pertinent to imaging-based biomarkers. If severe astrocytosis is a feature of ADNC + LATE-NC + HS-A, then specialized brain scanning methods may help indicate the presence of this pathologic pattern in the clinical setting. Clinical neuroimaging that predicts astrocytosis has been useful in some studies of epilepsy-linked HS (49–51). Further, radiographic studies of astrocytosis (e.g. as operationalized by T2-weighted and FLAIR signal on MRI, or other measures) have also been suggested to be a helpful resource in predicting neuropathologies in dementia (52–55). This approach may complement other imaging findings linked to LATE-NC + HS-A (56–60). In future studies, we hope to see more comprehensive correlations between the astrocytosis in HS-A and neuroimaging findings.

There were pitfalls and limitations to this study. For the pathology-defined groups, we included subjects with a substantial amount of ADNC because those are the usual contexts where LATE-NC + HS-A is observed; additional studies in “pure” LATE-NC (with and/or without HS-A) may provide additional insights. It would be challenging to develop groups of cases corresponding to all of the possible cut-points for the subtypes of ADNC, LATE-NC, and HS-A severities. Other subtypes of common brain pathologies, such as vascular pathologies and Lewy body diseases, could also be included but again could generate many further subsets of cases.

We also note that all of the subjects included in the current study were Caucasians. Although published studies have not described differences in the prevalence of LATE-NC with or without HS-A in different ethnic groups, some of the relevant genetic risk factors are differently expressed according to geographic heritage (61). Future work is required in more ethnically and racially diverse cohorts, which may show different results.

In summary, in cases of severe ADNC where either HS-A or TDP-43 pathologies were detected, the astrocytosis tended to be less severe than in cases with the combination of pathologies comprising ADNC + LATE-NC + HS-A. Severe ADNC by itself was associated with only marginally increased astrocytosis in CA1 and subiculum, versus low-pathology controls. A fundamental unanswered question is whether the excess GFAP+ astrocytosis seen in ADNC + LATE-NC + HS-A is exclusively reactive, or if the astrocytes may be contributory to the pathogenesis.
